# Different apoptosis pathways in *Leishmania* parasites

**DOI:** 10.1038/s41420-018-0092-z

**Published:** 2018-08-20

**Authors:** Louise Basmaciyan, Nadine Azas, Magali Casanova

**Affiliations:** 1 UMR PAM A, Valmis Team, 2 rue Angélique Ducoudray, BP 37013, 21070 Dijon Cedex, France; 2 Aix Marseille Univ, IRD, AP-HM, SSA, VITROME, IHU-Méditerranée Infection, Marseille, France

**Keywords:** Parasitic infection, Apoptosis

Flagellated parasitic protozoa of the *Leishmania* genus are responsible for the neglected tropical disease known as leishmaniasis. This worldwide disease causes between 20,000 and 30,000 deaths per year in about 97 countries (Global Health Observatory data from the World Health Organization, September 22, 2017). Available treatments are limited owing to toxicity, mode of administration, cost, and drug resistance. In response to various stimuli, *Leishmania* cells present a phenotype similar to that of apoptotic mammalian cells. Cell rounding up can be observed, as well as chromatin condensation, oligonucleosomal DNA fragmentation, and mitochondrial depolarization^1^. Since the term “apoptosis” describes a type of cell death defined by its morphological aspects according to the Nomenclature Committee on Cell Death^2^ and these features are encountered in *Leishmania*, as cited above, we can talk about not only cell death but also apoptosis in this unicellular parasite. Physiologically, apoptosis has been described in the parasite as a selfish altruism, regulating parasite densities within the vector and the mammalian host, and avoiding hyperparasitism^3^. Apoptosis in *Leishmania* may also permit successful infection by modulating host immunity^3^.

Classically, two main apoptotic pathways are described in mammalian cells: (i) the extrinsic pathway that is activated by recognition between an extracellular ligand and a death receptor and (ii) the intrinsic pathway activated by intracellular signals, which involves the mitochondrion and pro-apoptotic and anti-apoptotic molecules. Both pathways lead to a cascade of activations of specific proteins known as caspases, which are cysteine proteases. In *Leishmania*, the apoptotic pathway remains largely unknown, due to the lack of knowledge concerning the proteins involved, since the classic mammalian key proteins of apoptosis are not found in this parasite. For example, no death receptor has been identified in *Leishmania*^4^ and the presence of pro-apoptotic and anti-apoptotic molecules is still a matter of debate^5^. Previously, we studied the metacaspase LmjMCA, a cysteine peptidase present in *L. major* that shares similarities with caspases but has different substrate specificity^6^. We showed that LmjMCA is involved in apoptosis induced by the anti-*Leishmania* drug miltefosine, having a role similar to that of caspases. The purpose of this study is to investigate whether apoptosis in *Leishmania* consistently involves LmjMCA, regardless of the stimulus. To do so, we tested five molecules: four anti-*Leishmania* drugs (amphotericin B, curcumin, miltefosine, and pentamidine) and another molecule (H_2_O_2_). We recently showed that all these five molecules induce *L. major* apoptosis^7^.

**Fig. 1:**
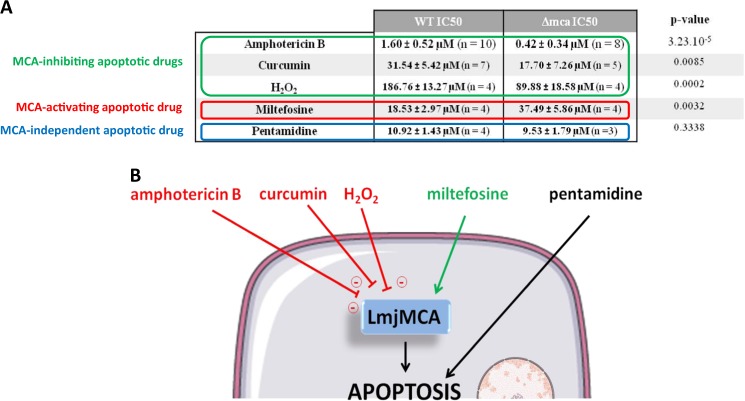
Different apoptosis pathways in *Leishmania* cells. (**A**) In vitro antileishmanicidal activity of different drugs/molecules on WT and LmjMCA-deficient (Δmca) *L. major* cells. The IC50 of WT cells and Δmca *L. major* cells are presented as mean of n experiments ± standard deviation. For statistical analysis, unpaired *t*-tests were done comparing the Δmca strain values with the WT strain values (BioStaTGV). The *p*-value of the different tests is written is the last column. (**B**) Summary of the different apoptosis pathways in *Leishmania*, regarding LmjMCA.

To assess whether the apoptotic pathway induced by the different molecules tested involved LmjMCA, we carried out a methyl thiazol tetrazolium (MTT) assay with the five molecules on the WT strain and on the LmjMCA-deficient *L. major* strain. We calculated the inhibitory concentration 50 (IC50), i.e., the molecule concentration inhibiting 50% of cell growth in comparison with a control without any molecule. As shown in Fig. 1a, we observed that the IC50 was significantly lower in the LmjMCA-deficient strain (Δmca) than in the wild-type (WT) strain for amphotericin B (0.42 and 1.60 µM, respectively), for curcumin (17.70 and 31.54 µM, respectively), and for H_2_O_2_ (89.88 and 186.76 µM, respectively). This suggests that amphotericin B, curcumin, and H_2_O_2_ induce LmjMCA inhibition. In contrast, the IC50 of miltefosine was significantly higher in the LmjMCA-deficient strain compared to the WT strain (37.49 and 18.53 µM, respectively), while no significant difference was observed between these two strains concerning pentamidine IC50 (about 10 µM). As a consequence, the miltefosine apoptotic pathway seems to involve LmjMCA activation, while the apoptotic pathway induced by pentamidine did not involve LmjMCA.

These results showed that the five molecules had different effects on LmjMCA. However, as recommended by Carmona-Gutierrez et al.^8^, “neologisms should be introduced with care and only when the characterization of a lethal process that bears new functional and biochemical aspects requires it. Otherwise, new expressions should be avoided to limit confusion”. Consequently, we prefer to talk of different apoptosis pathways induced by the different molecules rather than different types of cell deaths. More precisely, we identified three apoptosis pathways drawn in Fig. 1b, concerning LmjMCA: (i) an apoptosis pathway in which LmjMCA is activated (induced by miltefosine); (ii) a pathway in which LmjMCA is inhibited (amphotericin B, curcumin, and H_2_O_2_); and (iii) an LmjMCA-independent apoptosis pathway (pentamidine). In mammals, apoptosis is classically described as involving caspase activation. Hence, miltefosine would appear to induce the classic form of apoptosis. In 1996, Xiang et al.^9^ gave the first clear demonstration of caspase-independent regulated cell death. Since then, several articles have shown that regulated cell death can occur in the complete absence of caspases (reviewed in Bröker et al.^10^). In this case, several other proteases have been suggested to be involved, such as calpains, cathepsins, and serine proteases. In *Leishmania*, non-caspase proteases have been suggested to be involved in the cell death of the parasite, as the cysteine proteases calpains as reviewed in Branquinha et al.^11^. Moreover, El-Fadili et al.^12^ demonstrated that the cathepsin B-like enzyme LmjCPC is involved in *L. major* cell death. The third apoptosis pathway underlined in this article appears to be more original: the apoptosis pathway in which LmjMCA is inhibited. Two hypotheses can be formulated concerning this pathway: (i) either LmjMCA inhibition directly induces apoptosis, which could be explained by the role of LmjMCA in the cell survival process autophagy^6^ and by the fact that inhibiting autophagy induces cell apoptosis in stress conditions^13^, or (ii) apoptosis induced by amphotericin B, curcumin, and H_2_O_2_ involves an LmjMCA-independent pathway, and the inhibition of LmjMCA is not the cause of apoptosis.

These results support other articles which have already suggested different apoptosis pathways in *Leishmania*. For example, Vergnes et al.^14^ demonstrated that antimonial and miltefosine induced different cell death pathways in *Leishmania*. Foucher et al.^15^ have shown that amphotericin B and miltefosine induced different morphologic phenotypes in *Leishmania*, which is highlighted by the absence of cell shrinkage with amphotericin B while all other apoptotic markers were found with this drug^7^. In conclusion, this article highlights multiple apoptotic pathways in *Leishmania* in response to the addition of different molecules, while apoptosis is essential for successful survival of the population and for parasite infectivity^3^. However, other studies must be conducted to demonstrate whether these pathways are physiologically encountered in *Leishmania* cells and to better understand the entire pathway. To do this, genetic tools must be used, such as mutants deficient in a protein, rather than pharmacological inhibitors that might block the activity of several factors/pathways. In all cases, the study of an ancestral eukaryote such as *Leishmania* will contribute towards a better understanding of the evolution of regulated cell death in eukaryotes in general. These results will also possibly help the design of efficient new drugs based on their mode of killing of *Leishmania* parasites. Finally, identifying different cell death pathways will enable the combination of different drugs inducing different pathways in order to avoid or delay the appearance of drug resistance.
